# Plant litter chemistry and associated changes in microbial decomposition under drought

**DOI:** 10.1128/mbio.00438-26

**Published:** 2026-04-29

**Authors:** Brian Chung, Shi Wang, Zhao Hao, Steven D. Allison, Ashish A. Malik

**Affiliations:** 1 Department of Earth System Science, University of California 657745, Irvine, California, USA; 2 Earth and Environmental Sciences, Lawrence Berkeley National Laboratory 1666, Berkeley, California, USA; 3 Department of Ecology & Evolutionary Biology, University of California 189205, Irvine, California, USA; 4 School of GeoSciences, The University of Edinburgh 70448, Edinburgh, United Kingdom; 5 School of Biological Sciences, University of Aberdeen, Aberdeen, United Kingdom; Georgia Institute of Technology, Atlanta, Georgia, USA

**Keywords:** plant litter, bacteria, fungi, drought, decomposition

## Abstract

Drought has consequences for microbial decomposition rates, including indirect effects through changes in plant litter chemistry. Here, we studied the impact of a decade-long drought on plant litter chemistry and microbial decomposition traits in a semi-arid ecosystem during an 18-month litter bag experiment. We investigated litter sourced from four conditions: grass and shrub vegetation under ambient and reduced precipitation. We hypothesized that litter chemistry drives microbial decomposition capabilities and enzyme activity due to vegetation differences and drought effects on litter chemistry. We found that carbohydrate-rich grass litter had a higher abundance of decomposition genes detected using metagenomics and enzyme activity than more recalcitrant shrub litter, which was richer in lignin and lipids; these patterns were related to substrate supply. Drought decreased some carbohydrate fractions in grass litter but did not change the lignin fraction in grass and shrub litter, suggesting that drought does not make litter more recalcitrant. Most decomposition genes and enzyme activities were not significantly affected by drought, thereby maintaining decomposition rates. Microbial community succession patterns—decreasing fungal abundance and increasing bacterial abundance with time—corresponded with decreasing chitin gene abundance and increasing peptidoglycan gene abundance over time, indicating microbial necromass recycling. We demonstrate minimal litter chemistry-mediated effects of drought but show significant changes in community composition and their decomposition capabilities over time, highlighting that complex microbial-chemical interactions under climate change can influence ecosystem-scale processes.

**IMPORTANCE:**

Climate change is causing more severe and frequent droughts in semi-arid ecosystems, affecting soil microbes breaking down plant litter. Our research focuses on understanding the less studied pathway of drought impact on microbes via changes in plant litter chemistry. Drought can alter the plant litter chemistry by changing the composition and physiology of plants, which can alter microbial decomposition and ecosystem-level carbon cycling. We investigated litter decomposition traits of microbial communities in grass and shrub litter under long-term drought. There were significant changes in litter chemistry under drought but no increase in lignin fraction. Despite this, microbial communities maintained their decomposition capabilities under drought, highlighting the ability of microbes to adapt and continue functioning. We also demonstrate unique microbial community succession patterns and dead biomass recycling, which can have implications for carbon cycling rates in the ecosystem. This study sheds light on the complex microbial interactions that affect ecosystem functioning under climate change.

## INTRODUCTION

Changes in precipitation regimes are projected to occur with climate change, with increasing drought having already been observed in semi-arid ecosystems across the globe such as in California, western South America, and the Mediterranean (1). Increasing evapotranspiration due to an increase in the co-occurrence of high temperatures and low precipitation (2) is leading to more severe droughts. Furthermore, the number of extreme precipitation events, including periods of low precipitation, is also projected to increase (3). In addition, changes in precipitation are not uniform at a regional scale (1), with arid and semi-arid ecosystems in southern California experiencing decreasing annual precipitation (4).

The increasing severity and frequency of droughts can have major effects on key ecosystem processes such as decomposition and the organisms driving it, mainly fungi and bacteria. The effects on decomposer microorganisms can be both direct—through changes in abundance of individual taxa, community composition, and traits for drought stress tolerance—and indirect—through changes in the plant litter chemistry (5–7). Microbial response to drought may not always be consistent and may vary across ecosystems (8) or climate histories (9, 10). Drought in semi-arid ecosystems decreases litter decomposition rates in some systems (11–13), but not others (14). Decreases in decomposition have been attributed to decreases in microbial biomass (13) and the efficiency of extracellular enzymes (15). While drought shifted the overall microbial community composition of an oak forest toward fungal dominance, drought increased both bacterial and fungal abundance in a mixed pine-oak forest such that fungal:bacterial ratios remained unchanged (14). Drought can also shift investment in microbial traits such that allocation toward stress tolerance traits reduces decomposition capabilities.

Litter chemistry can be a major control on decomposition (16, 17), including decomposition in grasslands (18). Drought has been shown to alter litter chemistry (13, 19, 20) through changes in plant physiology (20) and changes in plant community composition (21, 22). These changes in litter chemistry, in turn, affect the microbial community, altering its composition by decreasing the bacterial abundance (13) and decreasing investment in extracellular enzyme activity by decreasing the proportions of certain litter substrates (15). Therefore, drought can exert direct and indirect effects on litter decomposition, and the indirect effects remain understudied (7).

Here, we investigate the effects of a decade-long drought on the decomposition traits of microbial communities during an 18-month litter bag experiment in a semi-arid ecosystem, specifically focusing on microbial resource acquisition traits, as influenced by the vegetation type and the corresponding litter chemistry. We tested the impact of drought on plant litter derived from grass and shrub vegetation that experienced either ambient or reduced precipitation for 10 years. We hypothesized that shrub litter decays more slowly than grass litter because it contains more lignin/lipids and less cellulose/hemicellulose, while drought alters the chemistry of both litter types, making them more recalcitrant, such that they decay more slowly. Further, we hypothesized that litter chemistry drives microbial decomposition capabilities and enzyme activity, whether due to vegetation differences or drought effects on chemistry. We tested these hypotheses using Fourier transform infrared spectroscopy (FTIR) to study changes in litter chemistry, shotgun metagenomics to measure decomposition capabilities as functional gene abundance, and fluorometric extracellular enzyme assays to quantify decomposer activity. We synthesize the knowledge to present evidence of the less-studied effects of drought via changes in plant litter chemistry on microbe-mediated decomposition rates, a key ecosystem function.

## RESULTS

### Shrub litter is more recalcitrant than grass litter

**Fig 1:**
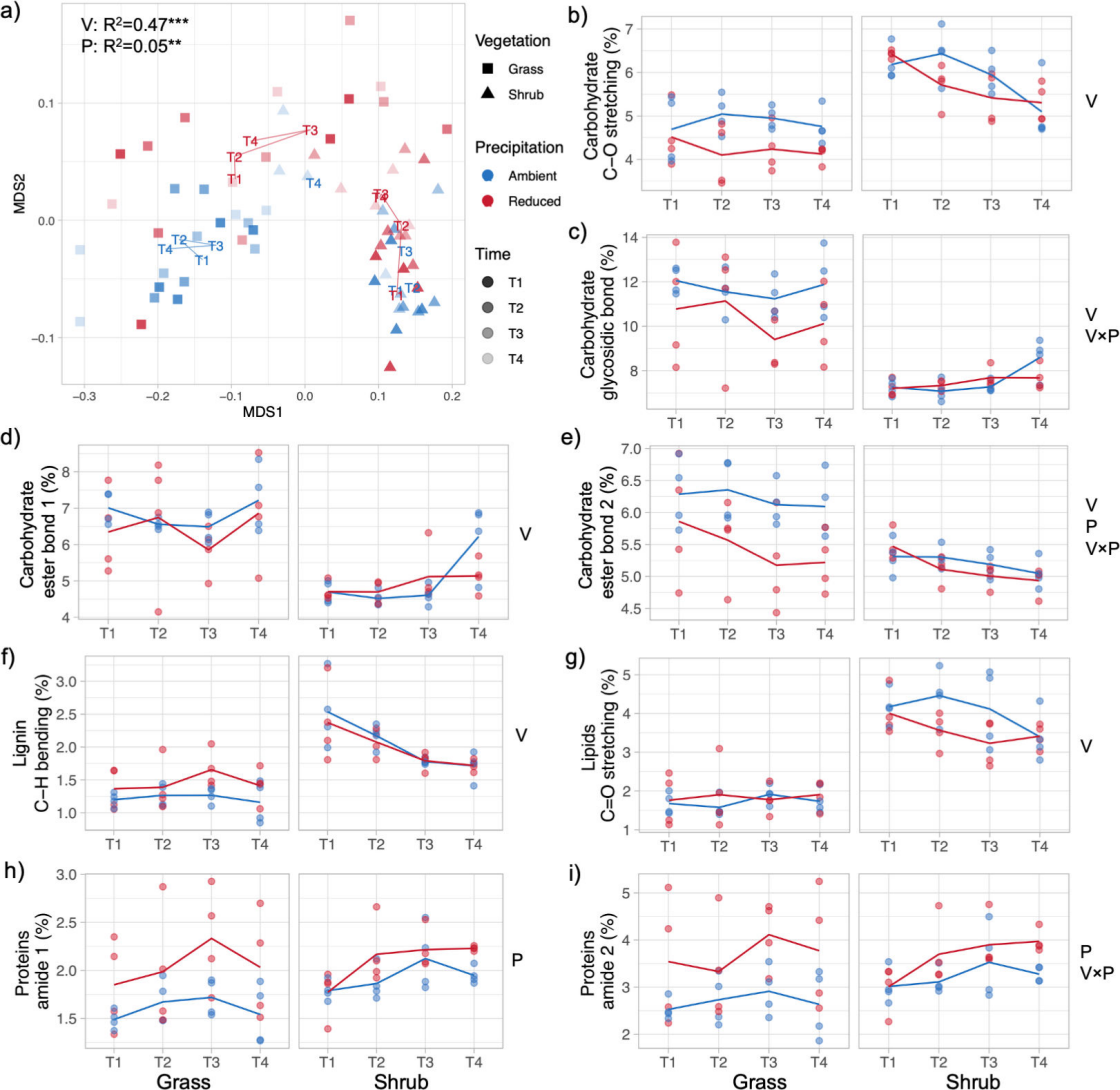
Litter chemistry differences over time across vegetation and precipitation treatments: (**a**) a non-metric multidimensional scaling (NMDS) plot of litter chemical composition derived from FTIR with PERMANOVA *R*^2^ and asterisks indicating significant vegetation (V) or precipitation (P) treatment effects (***P < 0.001, **P < 0.01, and *P < 0.05). T1 and T2 represent sampling points at the end of the first dry and wet season, respectively; T3 and T4 represent sampling points at the end of the second dry and wet season, respectively. Centroids derived from the four replicates at each time point are shown using text labels, and lines connecting consecutive time points show temporal patterns. (**b–i**) Changes in key compound classes over time for the four litter types; y-axis representing the proportional abundance estimated as the area under the curve assigned to a specific FTIR spectral range, with the letters at the side only showing significant vegetation (V), precipitation (P), or interaction effect derived using linear mixed-effect (LME) models and Tukey’s *post hoc* test. (**b**) Carbohydrate C-O stretching; (**c**) C-O deformation of glycosidic bonds; (**d**) carbohydrate ester bond 1; (**e**) carbohydrate ester bond 2; (**f**) C-H deformations in methyl and methylene groups in lignin; (**g**) lipid C=O stretching; (**h**) amide 1; (**i**) amide 2.

Litter chemistry differed between vegetation types (Fig. 1a), with grass litter generally having higher carbohydrate content than shrub litter. While shrub litter had a higher spectral area of carbohydrate C-O stretching (Fig. 1b; Tukey’s *post hoc P* < 0.001), grass litter had higher spectral areas of other carbohydrate features such as glycosidic bonds and carbohydrate esters (Fig. 1c through e; Tukey’s *P* < 0.001). The carbohydrate ester spectral area (Fig. 1e) is likely associated with hemicellulose (23), indicating that grass litter has higher proportions of hemicellulose than shrub litter. Shrub litter has a higher spectral area associated with C-H bending of methyl and methylene deformation (Fig. 1f; Tukey’s *P* < 0.001) and C=O stretching (Fig. 1g; Tukey’s *P* < 0.001). The observed C-H methyl and methylene deformation is characteristic of lignin (23), while the ester C=O stretching is characteristic of lipids (24). These results indicate that shrub compared to grass litter had higher proportions of more recalcitrant compounds, namely, lignin and lipids (Fig. 1f and g), indicating that shrub litter is more recalcitrant than grass litter, consistent with our hypothesized difference in decay rates between these two litter types.

### Drought had stronger effects on the chemistry of grass than shrub litter

While drought significantly affected litter chemistry of both litter types, as shown by an overall precipitation effect on some spectral bands (Table S1) and litter chemical composition (Fig. 1a; PERMANOVA *R*^2^ = 0.05, *P* < 0.01), drought had much stronger effects on grass litter than shrub litter (Fig. 1a, c, d and i). There were significant interactions between vegetation type and precipitation for carbohydrate glycosidic bonds and carbohydrate ester bond 2 (Fig. 1c and e; Table S1; Tukey’s *P* < 0.05). Drought lowered spectral areas in these two ranges in grass litter, but not shrub litter (Fig. 1c and e), indicating that drought only lowered carbohydrate content in grass litter. This is consistent with the smaller effect size of precipitation treatment on litter chemistry (PERMANOVA *R*^2^ = 0.05) in comparison to the effect size of vegetation type (PERMANOVA *R*^2^ = 0.47; Fig. 1a). While drought lowered some spectral areas associated with carbohydrates in grass litter (Fig. 1c and e), drought did not lower other carbohydrate features in grass litter, as indicated by the lack of significance under linear mixed-effect (LME) models (*P* ≥ 0.284; Fig. 1b and d). Similarly, drought increased protein concentration in litter of both types (Fig. 1h and i; Table S1; Tukey’s *P* < 0.001), although the effects were stronger for grass litter than shrub litter (Fig. 1i; LME *P* = 0.080, Tukey’s *P* < 0.01). Drought did not affect lignin or lipids in either litter types (Fig. 1f and g; Table S1; LME *P* ≥ 0.188), indicating that drought did not increase the recalcitrance of either litter types. This is inconsistent with the effects of drought predicted by our hypothesis.

### Decomposition genes not strongly affected by drought

**Fig 2:**
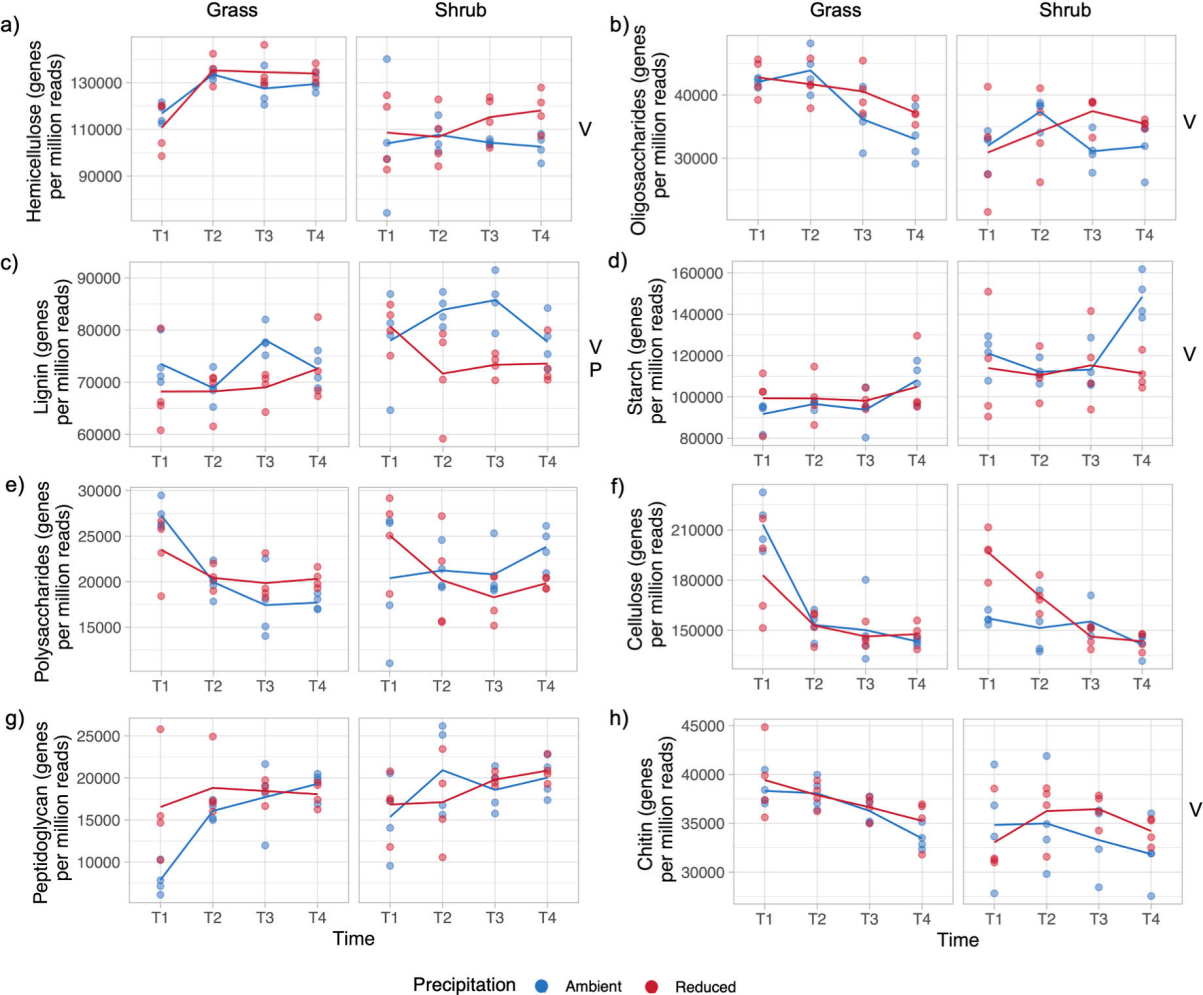
Gene-level decomposition capabilities changing over time across vegetation and precipitation treatments: CAZyme gene abundance for putative substrates. (**a**) Hemicellulose, (**b**) oligosaccharides, (**c**) lignin, (**d**) starch, (**e**) polysaccharides, (**f**) cellulose, (**g**) peptidoglycan, and (**h**) chitin. The letters at the side only show significant vegetation (V), precipitation (P), or interaction effects derived using linear mixed-effect models and Tukey’s *post hoc* test. T1 and T2 represent sampling points at the end of the first dry and wet season, respectively; T3 and T4 represent sampling points at the end of the second dry and wet season, respectively.

The abundance of carbohydrate active enzyme (CAZyme) genes for metabolizing carbohydrates—hemicellulose and oligosaccharides—was higher in grass litter than shrub litter (Fig. 2a and b; Tukey’s *P* < 0.001), while CAZyme genes for lignin were more abundant in shrub litter (Fig. 2c; Tukey’s *P* < 0.001). These differences in CAZyme gene abundance, when comparing broadly between the two litter types, are consistent with the differences in litter chemistry. The results across precipitation treatments do not support the indirect effects of drought through plant litter chemistry changes that we hypothesized. CAZyme genes for carbohydrates—hemicellulose, oligosaccharides, starch, polysaccharides, and cellulose—were not affected by drought in either of the litter types (Fig. 2a, b and d through f; Table S1; LME or Tukey’s *P* ≥ 0.152) whether drought decreased some carbohydrate fractions—as in grass litter—or not—as in shrub litter (Fig. 1c and e; Table S1). Drought decreased lignin-related genes across both systems (Fig. 2c; Tukey’s *P* < 0.01) despite drought not affecting lignin fractions in litter (Fig. 1f; Table S1).

### Patterns of community succession with decomposition

**Fig 3:**
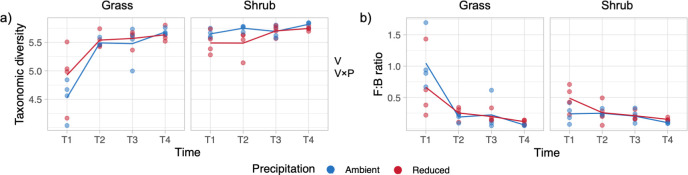
Diversity changes over time across vegetation and precipitation treatments: (**a**) Taxonomic diversity presented as alpha diversity based on genus-level annotations derived from metagenomics reads. (**b**) Fungal:bacterial ratios estimated as read abundance ratios of the two groups from the same data set. The letters at the side only show significant vegetation (V), precipitation (P), or interaction effects derived using linear mixed-effect models and Tukey’s *post hoc* test. T1 and T2 represent sampling points at the end of the first dry and wet season, respectively; T3 and T4 represent sampling points at the end of the second dry and wet season, respectively.

CAZyme gene abundances related to oligosaccharides, polysaccharides, and cellulose decreased over time (Fig. 2b, e and f), while CAZyme gene abundances related to hemicellulose and starch increased over time (Fig. 2a and d), indicating a succession of the decomposition of different substrates. These chemical changes were also linked to changes in microbial diversity (Fig. 3a) and composition (Fig. S2) over time across both litter types, with grass litter experiencing stronger changes and with temporal changes being stronger over the first wet season (T1 to T2; Fig. 3; Fig. S2). Taxonomic diversity increased over time in both systems (Fig. 3a), while fungal:bacterial ratios decreased over time in both systems (Fig. 3b). These changes in composition also corresponded with temporal trends in CAZyme genes involved in microbial cell wall metabolism. Gene abundance for peptidoglycan metabolism increased over time (Fig. 2g), while chitin gene abundance decreased over time in both systems (Fig. 2h).

### Extracellular enzyme activity is driven by substrate supply

**Fig 4:**
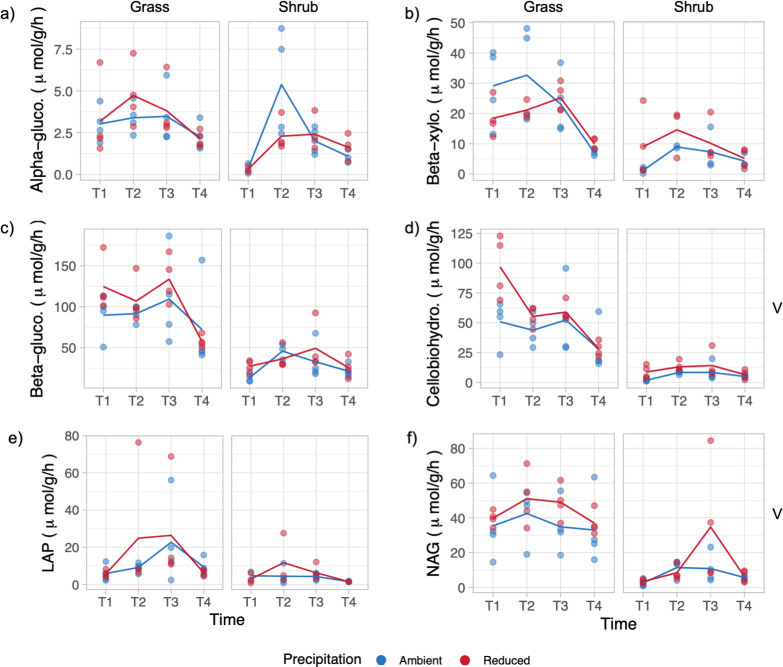
Extracellular enzyme activity measured as enzyme *V*_max_ for (**a**) ɑ-glucosidase, (**b**) β-xylosidase, (**c**) β-glucosidase, (**d**) cellobiohydrolase, (**e**) leucine aminopeptidase, and (**f**) N-acetyl-β-glucosaminidase. The letters at the side only show significant vegetation (V), precipitation (P), or interaction effects derived using linear mixed-effect models and Tukey’s *post hoc* test. T1 and T2 represent sampling points at the end of the first dry and wet season, respectively; T3 and T4 represent sampling points at the end of the second dry and wet season, respectively.

Enzyme activity tended to be higher in grass than shrub litter (Fig. 4), with statistically significant differences for the enzymes cellobiohydrolase and N-acetyl-β-D-glucosaminidase (Fig. 4d and f; Table S1; Tukey’s *P* < 0.001) and insignificant differences for ɑ-glucosidase (LME *P* = 0.799), β-glucosidase (LME < 0.001; Tukey’s *P* = 0.516), and β-xylosidase (LME *P* = 0.651) (Fig. 4a through c; Table S1). The higher carbohydrate content of grass litter (Fig. 1c through e) corresponded with larger pools of extracellular enzymes that target carbohydrates (Fig. 4a through d), suggesting that enzyme activity is driven by substrate supply rather than microbial demand.

Drought had no statistically significant effect on the activity of any enzymes, either as a main effect or as an interaction with vegetation (Fig. 4; Table S1). There was also very high variability across replicates, which could have obscured treatment effects. This result does not support the indirect effect of drought on enzyme activity that we hypothesized as activity of carbohydrate enzymes remained unchanged under drought (Fig. 4a through d) whether some carbohydrate fractions decreased—as in grass litter—or remained unchanged—as in shrub litter (Fig. 1c and e).

## DISCUSSION

### Induction of enzymes by their substrates

Resource acquisition traits and litter chemistry differed between vegetation types, as predicted by our hypothesis. Differences in lignin and carbohydrates between the vegetation types are consistent with those of other studies that compared one or two grass or shrub litter species (25, 26). We observed higher CAZyme gene abundance and carbohydrate-degrading enzyme activity in grass litter than in shrub litter (Fig. 2a and d and 4a through d). This explains the faster decomposition rates of grass compared to shrub litter in this experiment; mass loss in the shrub litter was 39.6% ± 3.9% compared to 52.1% ± 5.5% in grass litter after 14 months since the start of the field incubation (27). Because grass litter tended to have higher carbohydrate content than shrub litter (Fig. 1c through e), these results are consistent with the theory of induction of enzymes by their substrates (28, 29) and positive associations between CAZyme genes and their substrates that have been observed elsewhere (30, 31). Higher carbohydrate-degrading enzyme *V*_max_ in the grass community (Fig. 4a through d) could also stem from differences in microbial genomic content, with the grass microbial community having a higher abundance of hemicellulose and oligosaccharide CAZyme genes than the shrub community (Fig. 2a and b).

Grass litter had lower proportions of recalcitrant compounds such as lignin than shrub litter (Fig. 1f), which could also allow for greater enzyme *V*_max_ in grass litter (32). Microbes that specialize in lignin degradation possess more genes that function in cell signaling pathways rather than hydrolytic enzymes (33). Lignin also adsorbs hydrolases (34, 35), likely decreasing enzymatic breakdown and reducing the concentrations of substrates and intermediate degradation products that induce enzyme production (28). Both factors could be further contributing to the gaps in *V*_max_ between the shrub and grass litter microbial communities. In our study, chemical recalcitrance likely plays the most important role in determining the rates of decomposition.

### Microbial succession and necromass recycling

Along with broad differences in functional gene abundances between the litter communities, we also observed changes that correspond with succession in microbial communities as decomposition progressed. Grass litter community composition changed over the first wet season (T1 to T2) but appeared to stabilize afterward (Fig. 3; Fig. S2). This is consistent with the rapid initial changes in litter microbial community composition observed elsewhere (36, 37). Decreasing fungal-bacterial ratios with time (Fig. 3b) corresponded with increasing peptidoglycan gene abundance and decreasing chitin gene abundance (Fig. 2g and h), indicating microbial communities depolymerizing fungal and bacterial necromass as a carbon source. Some of the peptidoglycan genes we observed are used by bacteria to recycle their cell walls in the process of cell growth (38), which could also explain the increase over time. Decreasing chitin gene abundance is consistent with decreasing abundance of bacteria that decompose fungal cell walls (37) as well as decreasing fungal abundance. These opposing temporal trends in peptidoglycan and chitin gene abundance indicate a succession in microbial necromass recycling. Decreasing fungal abundance also corresponded with decreasing trends of β-glucosidase, β-xylosidase, and cellobiohydrolase *V*_max_ over time (Fig. 4b through d), trends that have been observed in a temperate oak forest (36, 37). Some studies show that fungi are the main producers of extracellular enzymes (39), and a previous study conducted in our grassland system found that the most abundant fungal taxa explained more variation in extracellular enzyme activity than the most abundant bacterial taxa (40).

### Effects of drought through litter chemistry changes were minimal

We did not observe support for our hypothesis on the indirect effects of drought through litter chemistry changes. In contrast to our predictions, drought did not increase the recalcitrance of either litter type as lignin remained unchanged under drought (Fig. 1f). Observations on the effect of drought on lignin have been mixed. While some studies found that litter that originated from drought environments had higher lignin than litter from ambient environments (13, 41), other studies showed that drought decreased lignin in litter of some, but not all plant species (42). In contrast to our predictions, resource acquisition trait values generally did not change (Fig. 2 and 4; Table S1) whether litter chemistry changed under drought—as in grass litter—or was unaffected by drought—as in shrub litter (Fig. 1; Table S1). Previous studies have shown negative correlations between lignin fractions and decomposition rates (16–18), and lignin has also been shown to decrease decomposition rates of specific litter fractions such as cellulose and hemicellulose (32). The lack of change in lignin under drought likely contributed to a lack of change in substrate availability, which explained the lack of response of resource acquisition traits to changes in litter chemistry under drought. Drought decreased lignin genes (Fig. 2g) despite not affecting litter lignin fractions (Fig. 1f). While litter lignin content can influence lignin gene abundance, these results indicated that drought directly decreased lignin genes in this study rather than indirectly by changing lignin content.

While the decreases in carbohydrate content in grass litter under drought are consistent with those of a previous study in this same field experiment (13), overall effects of drought on carbohydrate content in both vegetation types were small. Drought did not affect carbohydrate content in shrub litter (Fig. 1b through e) and therefore did not influence substrate availability. Substrate availability in soil is limited by substrate diffusivity, while substrate availability in litter likely is not (43), making it plausible that substrate availability in litter remains high even under low moisture conditions (44, 45). Our results suggest that grass litter chemistry might not have changed enough under drought to decrease substrate availability and investment in resource acquisition traits, while the lack of change in shrub litter chemistry under drought made it even less likely for substrate availability to change in shrub litter. Indeed, decomposition rates measured as mass loss were not significantly lower under drought in both grass (50.5% ± 3.3% under reduced precipitation treatment compared to 53.8% ± 7.2% under ambient precipitation) and shrub (41.8% ± 1.2% under reduced precipitation compared to 37.3% ± 4.5% under ambient precipitation) (27). Taken together, results from this semi-arid ecosystem suggest that decomposition genes and enzyme activities were not significantly affected by long-term drought, thereby maintaining litter decomposition rates.

### Study limitations and future work

We specifically sampled the litter of plant species that are characteristic of the two vegetation types (grass and shrub) rather than species that are characteristic of each plot, and therefore, our litter chemistry data might only be indicative of changes in plant physiology under drought (19, 20, 41, 42) and did not account for changes in plant community composition such as shrub to grass conversions observed at our study site (21, 22). Drought was shown to change plant community composition in observational studies over time (46) as well as in field experiments (21, 22), with drought being a factor that drives vegetation type conversion from chaparral ecosystems to exotic grasslands in California (22, 46). Such changes in plant community composition must be included in future experiments as they can change the litter that microbes decompose, affecting microbial communities, their traits, and decomposition rates (47). Furthermore, while our litter chemistry results are broadly consistent with litter chemistry results in other studies (13, 19, 20, 25, 26, 48), it is difficult to tease apart certain litter fractions and their responses to drought with our FTIR-derived litter chemistry data. A more quantitative and higher-resolution method of analyzing litter chemistry changes might provide clearer results.

Consistent with our study, studies of litter decomposition in Mediterranean ecosystems so far indicate that drought-induced changes in litter chemistry either do not influence decomposition rates (13, 49) or do not influence decomposition rates as much as direct effects of drought (50). Plant litter chemistry can influence how microbial traits (8, 27) and decomposition rates (11, 12) respond to drought. Since our study indicates that microbial decomposition traits are resistant to drought-induced changes in litter chemistry following changes in plant physiology, microbial decomposition traits might be more likely to change if drought also changes plant community composition, especially if plant communities undergo type conversion.

## MATERIALS AND METHODS

### Field experiment design

This study took place at the Loma Ridge Global Change Experiment (33°44′N, 117°42′W, 365 m elevation) near Irvine, CA, USA. The climate is Mediterranean, with a cool rainy season from November to April and a warm dry season from May to October of each year. The mean annual temperature is 17°C, and the mean annual precipitation is 325 mm (13). We studied coastal sage scrub (i.e., shrub) and grassland (i.e., grass) plots subjected to reduced or ambient precipitation treatments (22). This design led to four treatment combinations (two vegetation types × two precipitation treatments). Each treatment combination had four replicate plots, for a total of 16 plots (four treatment combinations × four replicate plots). The reduced precipitation treatment plots were covered with clear polyethylene tarps during a subset of winter storms, reducing annual precipitation by ~40% (13, 22). Grass plots (6.7 m × 9.3 m) were dominated by exotic annual grasses of the genera *Avena*, *Bromus*, *Festuca*, and *Lolium* and forbs such as the genus *Erodium* (8). Shrub plots (18.3 m × 12.2 m) were dominated by the native shrubs *Salvia mellifera*, *Artemisia californica*, and *Malosma laurina* (22).

We measured decomposition rates, litter chemistry, metagenomics-derived functional gene abundance, and enzyme activity of plant litter at the field site with continued precipitation treatment (27). Plant litter was sampled on 30 August 2017 from all four replicate plots within each treatment combination. We only sampled litter from species that are representative of each vegetation type (i.e., only litter from shrub species was sampled from shrub plots of both precipitation treatments). Litter from all plots within each treatment combination was combined and mixed by hand while keeping treatment combinations separate from each other. We then made litter bags from 1-mm window-screen mesh and filled each bag with 6 g of litter from one treatment combination. Litter bags were deployed on 12 September 2017 and were collected from each plot over four time points. In total, this study deployed 64 litter bags (16 plots × four time points), with 16 litter bags (one litter bag from each plot) being collected at each time point for laboratory analysis. We collected litter bags on 30 November 2017 (T1), 11 April 2018 (T2), November 2018 (T3), and February 2019 (T4). An aliquot of the sampled litter was ground in a coffee mixer (a quick whirl for 5 s) to create a coarse powder, which was used for subsequent analyses.

### Litter chemistry

The chemical composition of the plant litter organic matter was measured using attenuated total reflection-Fourier transform infrared (ATR-FTIR) spectroscopy. The ground litter samples were gently pressed down on a clean surface of the germanium crystal in an ATR configuration (Smart Orbit; Thermo Fisher Scientific). Infrared light beamed from the interferometer (Nexus 870; Nicolet) was focused onto the interface between the sample and the top surface of the crystal through the lower facet. The sample spectrum was recorded with a spectral resolution of 4 cm^−1^ over the infrared range (4,000–600 cm^−1^). First, data were sum-normalized (peak area normalized to total peak area). Then, compositional differences along the entire spectrum were studied using principal component analysis (PCA) and non-metric multidimensional scaling (NMDS) in R using the vegan package (51), with visualizations created using ggplot2 (52). Spectral ranges that showed distinct variation across the treatments as observed using PCA (Fig. S1) were then quantified as peak area under the curve and assigned to different functional groups for different compound classes, as described in the literature (23, 24, 53).

### Metagenomics

DNA was extracted from a 50-mg aliquot of ground litter from all 64 samples using ZymoBiomics DNA isolations kits (Zymo Research, Irvine, CA, USA) following the manufacturer’s instructions. Sample homogenization was performed by bead beating for 5 min at the maximum speed of 6.0 m/s (FastPrep-24 High Speed Homogenizer, MP Biomedicals, Irvine, CA, USA). Gel electrophoresis, a Qubit fluorometer (LifeTechnologies, Carlsbad, CA, USA), and a NanoDrop 2000 spectrophotometer (Thermo Scientific, USA) were used to assess the purity and concentration of extracted DNA. Library preparation and metagenomic sequencing were carried out at the University of California Davis Genome Center. We used NovaSeq (Illumina, San Diego, CA, USA) with PE150 sequencing and the default insert size of 250–400 bp. Taxonomic classification up to genus level was performed using a reads-based assessment with the RefSeq database (maximum e-value cutoff of 10-5, minimum identity cut-of 60%, and minimum length of sequence alignment of 15 nucleotides) on Metagenomics Rapid Annotation using Subsystems Technology (MG-RAST) server version 4.0.3 (54).

We used Metagenome Orchestra (MAGO) (version V2.2b; 2020-03-08) (55) to produce metaSPAdes (version 3.13.0) (56) assemblies for individual samples. Within MAGO, the quality control of the paired-end reads was carried out with fastp (version v0.20.0) (57) to keep a Q30 read quality while carrying out adapter trimming. seqtk (version 1.3-r106) (58) was used to remove contigs shorter than 1,000 bp from the metaSPAdes assemblies. Contig-level data were used to assess community-aggregated functional differences across treatments. Prodigal (version 2.6.3) (59) was used to carry out gene-calling of metagenomic contigs from the individual sample assemblies, which was then queried against the carbohydrate active enzymes (CAZy) database using dbCAN2 (version 2.0.11) (60). PROKKA (version 1.13.7) (61) was run in the metagenome mode over the assemblies to generate respective annotations. To produce a community gene abundance table across the treatments, each data set of quality-controlled paired-end reads was aligned against its respective assembly using BWA (version 0.7.17-r1188) (62). SAMtools (version 1.9) (63) was used to convert the alignments to binary format as well as to sort them. HTSeq (version 0.11.2) (64) was employed to count the number of reads aligned to the annotated features by PROKKA across each sample. CAZy gene abundances were normalized by total protein-coding genes predicted using Prodigal. Normalization accounts for variation in sequencing depth and assembly bias to provide absolute count data. CAZyme genes for specific substrates (cellulose, hemicellulose, polysaccharides, lignin, starch, oligosaccharides, peptidoglycan, and chitin) were summed to obtain the total gene abundances linked to degradation of the substrates (65). Visualizations were made using ggplot2 (52).

### Extracellular enzyme assays

We performed extracellular enzyme assays on hydrolytic enzymes using previously reported fluorometric protocols (66, 67). Litter from each collected litter bag was homogenized in 25 mM maleate buffer with pH 6. The resulting homogenate was plated in 96-well opaque microplates with standards, controls, and serial dilutions of their respective substrates. Microplates were incubated at room temperature for 4 h, and fluorescence was then measured in a plate reader. Enzyme activity was then calculated from fluorescence data (67) and divided by the dry weight of the litter that was homogenized. The resulting enzyme activity was then plotted against substrate concentration in scatterplots using matplotlib (version 3.3.2) in Python. The scatterplots were manually inspected for the artifact of substrate inhibition, in which enzyme activity decreases at high substrate concentrations instead of approaching *V*_max_ due to the substrate now acting as an inhibitor (68, 69). Leaving these data points in model fitting can underestimate *V*_max_ (69). These data points were removed, and the resulting enzyme activity was fitted to the Michaelis-Menten equation using the curve_fit() function in scipy (version 1.5.2) to produce *V*_max_ in units of µM/g dry litter/h. *V*_max_ values from this curve-fitting were then subjected to further statistical analysis.

### Statistical analysis

Additional statistical analysis, on top of PERMANOVA and PCA on litter chemistry and visualizations, was conducted in Python (version 3.8.5). Linear mixed-effect modeling—conducted using the package statsmodels (version 0.12.0)—was performed on percent FTIR spectral areas of specific bands, CAZyme gene abundance, and *V*_max_ values, with vegetation, precipitation, and their interaction as fixed effects and the collection time point—in days since deployment—and plot as random effects. Residuals were checked for normality after each model fit using the Shapiro-Wilk test from scipy, and the dependent variable was transformed by log_10_, reciprocal, or square root transformations and refitted until the model with the most normal residuals—having the largest Shapiro-Wilk *P*-value—was produced. The square root transformation was dropped as it often did not produce the model with the most normal residuals. Tukey’s pairwise comparisons were performed as a *post hoc* test on levels of main effects and combinations of their interactions that were statistically significant in linear mixed-effect models (*P* < 0.05). Cohen’s *D* was calculated as a measure of effect size for statistically significant main effects (Table S1).

## Data Availability

The sequencing data set generated and analyzed in the current study is available in the NCBI Sequence Read Archive through BioProject number PRJNA1178105 with accession numbers from SRR31127223 to SRR31127332.

## SUPPLEMENTAL MATERIAL

File S1 mbio.00438-26-s0001.xlsx FTIR raw peak heights per wavelength.

File S2 mbio.00438-26-s0002.xlsx FTIR total peak area per substrate.

File S3 mbio.00438-26-s0003.xlsx Taxon counts.

File S4 mbio.00438-26-s0004.xlsx Taxonomic annotations.

File S5 mbio.00438-26-s0005.xlsx CAZy read counts total by substrate.

File S6 mbio.00438-26-s0006.xlsx CAZy read counts for all gen.

File S7 mbio.00438-26-s0007.xlsx Sample labels.

Supplemental material mbio.00438-26-s0008.pdf Fig. S1 and 2 and Table S1.

Captions mbio.00438-26-s0009.pdf Supplemental file captions.

ASM does not own the copyrights to Supplemental Material that may be linked to, or accessed through, an article. The authors have granted ASM a non-exclusive, world-wide license to publish the Supplemental Material files. Please contact the corresponding author directly for reuse.
