# Supplementary material for: Plant litter chemistry and associated changes in microbial decomposition under drought
Source: mBio. 2026 Apr 29;17(6):e00438-26. doi: 10.1128/mbio.00438-26 (PMC13251422; doi:10.1128/mbio.00438-26)

Supplementary Information

Figure S1: FTIR-derived spectral abundance for the range of wavelengths and their principal components (PCs) from a principal component analysis of all samples were used to identify the peaks that showed distinct variation across the treatments. The following wavelengths were used in our analysis of changes in plant litter chemistry based on literature.

970-1015: Carbohydrates, ester bonds 1 (Madari et al., 2006)
1015-1080: Carbohydrates, glycosidic bond (Madari et al., 2006; Zhuang et al., 2020)
1100-1160: Carbohydrates, ester bonds 2, likely hemicellulose (Zhuang et al., 2020)
1160-1230: Carbohydrates, C-O stretching (Madari et al., 2006; Zhuang et al., 2020)
1450-1475: Lignin, C-H bending, methyl/methylene deformation (Zhuang et al., 2020)
1545-1600: Proteins, amide 2, N-H bending (Madari et al., 2006)
1620-1645: Proteins, amide 1, N-H bending (Madari et al., 2006)
1700-1750: Lipids, aldehyde or esters with C=O stretching (Filley et al., 2008)

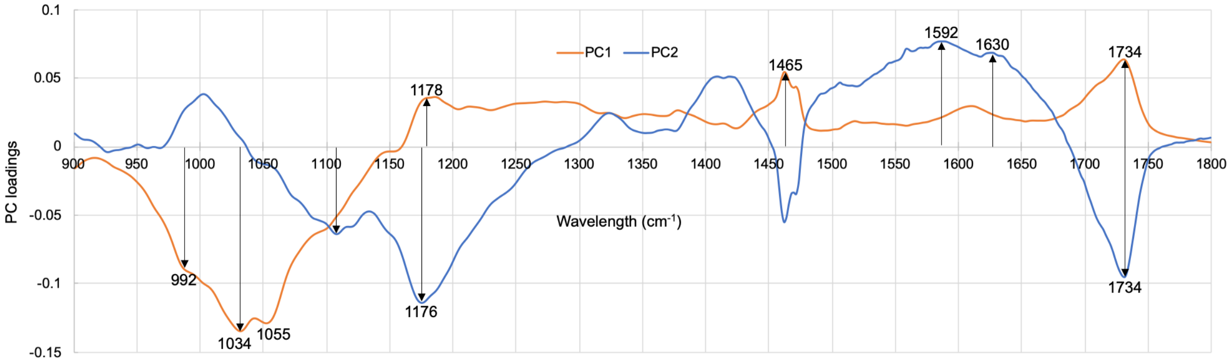

Table S1. Effects of vegetation communities and precipitation treatments on enzyme Vmax, FTIR litter chemistry, and CAZyme gene abundance. Cohen's D is calculated for significant main effects (either vegetation or precipitation) while significant vegetation and precipitation interactions are denoted with an asterisk. Insignificant results have empty cells. P-values are denoted for significant main effects and their interactions as asterisks * $p < 0.05$, ** $p < 0.01$, *** $p < 0.001$, with main effects using p-values from Tukey's pairwise comparisons and interactions using p-values from mixed effect models. P = Precipitation treatment. V = Vegetation community.
+ Not significant under mixed effect modeling but had groups that were statistically significantly different ($p < 0.01$) under Tukey's pairwise comparisons

		Transformation	V	P	V x P
Enzyme Vmax	AG	log10			
	AP	log10			
	BG	reciprocal			
	BX	log10			
	CBH	log10	2.9328***		
	LAP	reciprocal			
	NAG	log10	2.4994***		
FTIR band	1015 - 970 cm^{-1}	reciprocal	2.0241***		
	1080 - 1015 cm^{-1}		2.7182***		*
	1160 - 1100 cm^{-1}		1.2610***	0.7146**	*
	1230 - 1160 cm^{-1}		2.0109***		
	1475 - 1450 cm^{-1}	reciprocal	2.0938***		
	1600 - 1545 cm^{-1}			0.9759***	+
	1645 - 1620 cm^{-1}			0.8644***	

	1750 - 1700 cm^{-1}		3.4802***		
Putative substrate CAZyme gene abundance	Cellulose				
	Chitin		0.8150**		
	Hemicellulose		1.6573***		
	Lignin		1.0556***	0.7647**	
	Oligosaccharides	reciprocal	1.1331***		
	Peptidoglycan				
	Polysaccharides				
	Starch		1.3334***		
Community composition	F:B	reciprocal			
	Taxonomic diversity		0.9080***		+

Figure S2: Microbial community composition changes over time across vegetation and precipitation treatments presented using NMDS ordination. Taxonomic composition is based on genus-level annotations derived from metagenomics reads. T1 and T2 represent sampling points at the end of the first dry and wet season, respectively; T3 and T4 represent sampling points at the end of the second dry and wet season, respectively. Centroids derived from the four replicates at each time point are shown using text labels and lines connecting consecutive time points show temporal patterns.

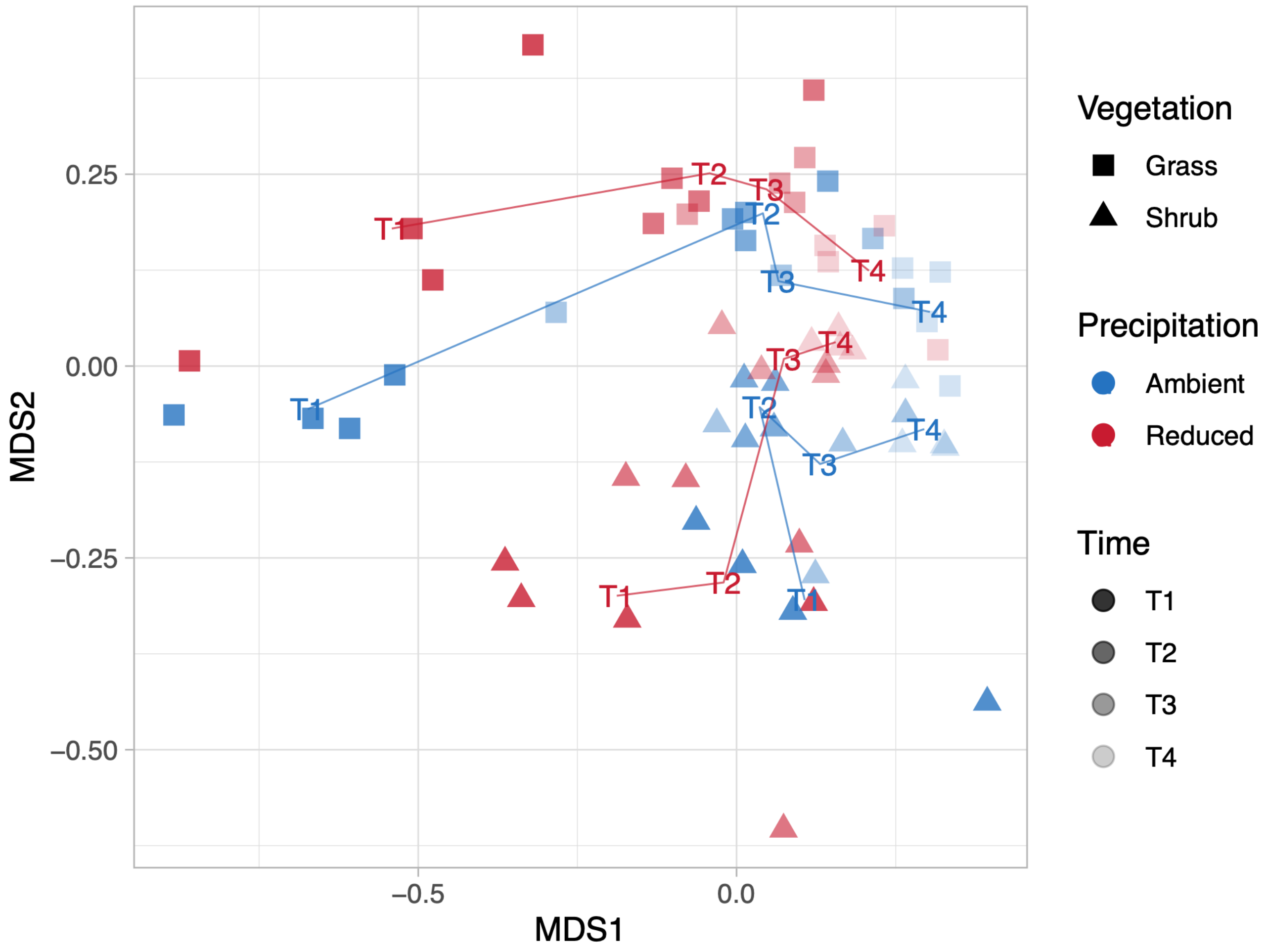

Supplement: Supplemental material — Fig. S1 and 2 and Table S1. [file mbio.00438-26-s0008.pdf]
