# Supplementary material for: Plant litter chemistry and associated changes in microbial decomposition under drought
Source: mBio. 2026 Apr 29;17(6):e00438-26. doi: 10.1128/mbio.00438-26 (PMC13251422; doi:10.1128/mbio.00438-26)
Supplement: Captions — Supplemental file captions. [file mbio.00438-26-s0009.pdf]

Supplemental files captions

Supplemental File 1: FTIR raw peak heights per waveleght recorded with a spectral resolution of 4 cm^{-1} over the infrared range (4,000-600 cm^{-1}).

Supplemental File 2: FTIR sum-normalized total peak area per spectral ranges that were assigned to different functional groups for compound classes.

Supplemental File 3: Taxa counts at the genus level performed using a reads-based assessment on MG-RAST.

Supplemental File 4: Taxonomic annotations up to genus level performed using RefSeq database on MG-RAST.

Supplemental File 5: CAZy read counts normalized by total protein-coding genes predicted using Prodigal.

Supplemental File 6: CAZy read counts for specific substrates obtained as the sum abundances of gene linked to degradation of particular substrates.

Supplemental File 7: Sample metadata with sample codes and IDs
